# The relationship between childhood trauma, personality, and subjective well-being in early and late adolescence: a network analysis

**DOI:** 10.1038/s41598-026-41659-0

**Published:** 2026-02-26

**Authors:** Weixi Zheng, Lian Zhou, Xin Lv, Jiayu Li, Yuhong Zhou

**Affiliations:** 1 New York University Shanghai, Shanghai, China; 2 Xiaguan No.1 Middle School, Dali, China; 3 Faculty of Psychology, Southwest University, Chongqing, China; 4 College of Education, Hebei Normal University, Shijiazhuang, China; 5 Psychological Research and Counseling Center, Southwest Jiaotong University, High-tech West Zone, Chengdu, 611756 China

**Keywords:** Childhood trauma, Personality, Subjective well-being, Adolescence, Network comparison, Health care, Psychology, Psychology

## Abstract

Childhood trauma is strongly associated with impaired subjective well-being (SWB) in adolescence. However, the underlying mechanisms, particularly how they differ across specific developmental stages, remain inadequately explored. Based on the stress process model, childhood trauma may shape adolescent SWB by influencing the development of personal resources, such as personality traits and self-esteem. This study used network analysis to investigate the relationships among these factors based on the stress process model and compared the network model differences between early and late adolescents. A total of 2,620 adolescents aged 12–18 participated in the study. The results showed that neuroticism and self-esteem exhibited consistently strong connections with SWB across age groups within the network models. Emotional abuse and emotional neglect were consistently negatively connected to positive personal resources such as agreeableness, conscientiousness, extraversion, and self-esteem across all age groups, while emotional abuse was strongly positively connected to negative personal resources like neuroticism, which in turn were linked to adolescents’ SWB. Additionally, the associations between personality traits and SWB differed by developmental stage. Specifically, early adolescents exhibited a stronger connection between extraversion and SWB, while late adolescents showed a stronger connection between agreeableness and SWB. Early adolescents also displayed a weaker connection between openness and self-esteem compared to late adolescents, indicating that differences in the relationships among personality traits across developmental stages may be important factors influencing SWB. This study provides valuable insights into the developmental stage-specific mechanisms linking childhood trauma, personality traits, and subjective well-being, offering a foundation for age-tailored interventions to enhance adolescent mental health.

**Supplementary Information:**

The online version contains supplementary material available at 10.1038/s41598-026-41659-0.

## Introduction

Subjective well-being (SWB) is people’s assessment of what is happening in their lives, which includes both reflective cognitive evaluations and emotional responses to life^1^, and is comprised of three components: life satisfaction, positive affect (PA), and negative affect (NA)^2^. Studies have found that SWB is associated with positive developmental outcomes in adolescence and that this positive protective effect persists into adulthood^3^, such that adolescents with high SWB experience more positive peer and family relationships, better academic performance, and lower depression and anxiety^4–8^, and less problematic social media use, substance abuse, and addictive behaviors^9–11^. It is evident that SWB is an important indicator of adolescent mental health^12,13^ and a predictor of the occurrence of psychological disorders^14^. Understanding the factors that influence SWB and the mechanisms through which it is shaped is essential for advancing adolescent mental health research and developing effective interventions.

### The relationship between childhood trauma and subjective well-being

Childhood trauma is a significant negative predictor of adolescents’ subjective well-being^15,16^. Defined as physical, emotional, or sexual abuse, as well as physical and emotional neglect by a caregiver or older individual^17,18^, childhood trauma is one of the most pervasive risk factors for poor mental health outcomes. Studies have consistently linked childhood trauma to lower life satisfaction^19,20^ and reduced positive affect, such as diminished hope, optimism, and self-efficacy^21^. Additionally, childhood trauma has long-lasting detrimental effects on SWB and mental health, affecting children, adolescents, and even adults^22,23^. Recent studies have demonstrated that childhood trauma is associated with an increased risk of depression, anxiety, and other psychological issues in adulthood^24,25^. These findings underscore the critical role of this early adverse experiences in shaping long-term mental health outcomes. Despite these established associations, the complex interplay between childhood trauma and SWB through a spectrum of personality traits, and how these pathways may differ across developmental stages, remains less explored.

### The relationship between childhood trauma and personality

The theoretical framework for this study is derived from the stress process model^26^. This model posits that the impact of stressors on mental health is primarily mediated through their effect on personal and social resources. Prior empirical work aligns with this premise, suggesting that childhood trauma is associated with compromised psychosocial resources (e.g., self-concept and self-esteem) and maladaptive personality functioning (e.g., higher neuroticism), which are relevant to later well-being outcomes^27–29^. The model suggests that childhood trauma can hinder the development of positive personal resources, while promoting the formation of negative resources. This integrative perspective on “resources” is strongly supported by the Conservation of Resources (COR) theory proposed by Hobfoll^30^, which defines resources as “those objects, personal characteristics, conditions, or energies that are valued by the individual”^30^. Within this framework, both broad personality traits and self-esteem are conceptualized as distinct but comparable personal characteristic resources, the former defined as resources that “generally aid stress resistance”^30^, and the latter explicitly listed as a key example of a resource^30^. This provides a unified theoretical basis for examining their shared role in the stress process. Specifically, within the framework of the Five-Factor Model (FFM) of personality^31^, traits such as high neuroticism, impulsivity, and borderline personality traits, are more prevalent in individuals who have experienced childhood abuse^28,32^. On the other hand, the development of adaptive personality traits, such as agreeableness, openness to experience, conscientiousness, and extraversion, are hindered by early trauma, which may limit an individual’s ability to effectively engage with others, regulate emotions, and adapt to challenges^33,34^, which may further impair the experience of subjective well-being. Furthermore, childhood trauma is associated with lower self-esteem^29,35^. And self-esteem is a core positive personal resource, reflecting a fundamental sense of self-worth that buffers against challenges. Therefore, it is evident that childhood trauma has a crucial factor on obstructing the development of positive personal resources and fostering the emergence of negative traits.

### The relationship between personality and subjective well-being

Personality has been shown to be one of the most reliable and powerful predictors of subjective well-being (SWB)^36^, with its influence extending over a decade or more in adolescents^37^. Prior studies consistently demonstrate that SWB correlates positively with positive personal resources. For example, previous research has found that high agreeableness and conscientiousness contribute to favorable interpersonal feedback and reinforcement, leading to elevated SWB^38^. Openness promotes SWB by fostering personal growth and appreciation for new experiences^39^, while conscientiousness enhances SWB through self-discipline and goal attainment^40^. Furthermore, self-esteem plays a significant role in SWB. High self-esteem enhances resilience and the ability to cope with adversity, leading to improved SWB^41–43^ and high self-esteem acting as a buffer that mitigates negative outcomes associated with adverse experiences^44,45^. Meanwhile, negative personal resources such as neuroticism was found to be negatively associated with SWB^46,47^. Individuals with high neuroticism tend to focus excessively on negative experiences, experiencing heightened negative emotions that impair SWB^48,49^.

### The group difference between early and late adolescence

Adolescence is a critical period of physical and psychological development, characterized by significant changes in neurocognitive, emotional, and personality domains as neural circuits gradually mature. Research in developmental neuroscience commonly distinguishes between different stages of adolescence, such as comparing early adolescents with mid-adolescents, to understand these transitions^50^. Consistent with this approach, this developmental phase is commonly divided into two stages: early adolescence (ages 12–15) and late adolescence (ages 16–18),a categorization that has been widely adopted in empirical adolescent research when testing age-group differences (e.g^51,52^.,. Early adolescence is marked by a second wave of synaptogenesis, followed by synaptic pruning as the brain matures, a process that continues and becomes more pronounced in late adolescence^53^. Beyond these normative neurodevelopmental changes, adolescence has been characterized as a sensitive window during which stressors can exert differential influences on neurodevelopment and behavior^54,55^. However, as a salient stressor, childhood trauma has shown inconsistent associations with later psychosocial outcomes across adolescent developmental stages: some studies suggest stronger effects in early adolescence, whereas others report more pronounced effects in late adolescence^56,57^, suggesting that trauma-related pathways may not be uniform across developmental periods. Accordingly, potential age-stage differences in the associations between childhood trauma and subsequent personal resources and SWB were examined in the present study in an exploratory manner.

Exposure to early life stress, such as family violence or emotional abuse, during critical developmental periods has been shown to profoundly and enduringly affect brain development^58^. For example, such stressors can disrupt the brain’s dopaminergic system, leading to reduced connectivity between the ventral tegmental area and hippocampus, potentially serving as an adaptive mechanism to prevent the overwriting of traumatic memories^59^. Additionally, early life stress accelerates the maturation of frontoamygdala connectivity, which may provide resilience but also increases the risk of developing psychopathology during adolescence^60–62^. From a personality development perspective, early adolescence often shows a temporary deceleration in personality maturation, that is, a normative and transient pattern of decreases in traits such as extraversion, conscientiousness, agreeableness, and openness, and an increase in neuroticism compared to earlier stages of development^63^. Investigating such developmental changes necessitates a framework that distinguishes between different stages of adolescence^50^. While personality traits begin to form early in life, they gradually stabilize over time^64^. Concurrently, self-esteem follows a normative developmental trajectory, tending to increase from adolescence to middle adulthood^65^.

In summary, the stress-induced alterations in adolescent brain development are posited to underlie the observed changes in personality formation. These personality traits, in turn, serve as key mechanisms through which early life stress ultimately influences subjective well-being. Considering the developmental characteristics of early and late adolescence, including the ongoing maturation of neural circuits, personality traits, and self-esteem, it is crucial to examine group differences in the relationships between childhood experiences, personality development, and SWB across these two stages. Given that early and late adolescence differ in the developmental context in which personality resources and self-evaluations operate, we expected that age-group differences would be reflected in the pattern and relative salience of associations among childhood trauma dimensions, personal resources (personality traits and self-esteem), and SWB.

### Network analysis and network comparison

Network analysis is a powerful analytical tool that visually represents the relationships between variables, providing valuable insights into complex systems^66^. In contrast to the correlation network model, the partial correlation network model divides nodes into distinct communities to illustrate which variables are more closely connected^67^. The edges between nodes represent the unique associations among them, with non-zero edges depicting connections after accounting for all other variables in the network. This approach can reveal more precise and meaningful correlations between variables^68,69^. This approach provides a systems-level perspective, allowing us to identify the most central variables and the specific connections between different clusters (e.g., trauma and personality), thus complementing findings from conventional methods. Compared with conventional approaches such as multiple regression or SEM, which typically require a priori specification of predictor–outcome roles, the network approach models all variables simultaneously and estimates unique associations (partial correlations) among nodes while controlling for the rest of the system. This allows us to (a) identify which trauma dimensions are uniquely linked to specific personality traits and SWB beyond bivariate correlations, and (b) characterize the relative importance of variables within the overall system (e.g., central nodes) without imposing a strict causal hierarchy. We emphasize that the network approach is complementary rather than a substitute for conventional methods: it provides a systems-level, data-driven description of interdependence that can inform and refine subsequent hypothesis-driven modeling.

A network comparison test further enhances this analysis by allowing researchers to examine whether and how subgroups within a population differ in terms of node connectivity. Specifically, it evaluates differences in network structure (i.e., the patterns of interconnections among nodes across subsamples), global strength (i.e., the overall strength of all edges in the network), and edge strength (i.e., the strength of specific connections between nodes)^70^. When network structures are found to be similar, it suggests that nodes interact in comparable ways across different groups. On the other hand, networks with higher global strength may indicate stronger feedback loops among nodes, which could correspond to heightened vulnerability to severe mental disorders^71^. Furthermore, analyzing differences in edge strength provides a deeper understanding of variations in specific associations between nodes^72^. Consistent with this approach, network analysis has been used in prior empirical studies to characterize multivariate relations among childhood adversity, personality-related characteristics, and mental health and developmental outcomes in youth and other populations (e.g^73–76^.,.

### The current study

According to the stress process model^26^, we examine how childhood trauma, as a chronic stressor, may impede the development of positive personal resources (e.g., adaptive personality traits, self-esteem) and foster negative resources (e.g., neuroticism), which in turn influence subjective well-being. In the present manuscript, “personal resources” is used as a theoretical (stress-process) umbrella term for relatively stable personal characteristics (e.g., personality traits and self-esteem) that condition stress exposure–outcome associations^26,30^. Importantly, the labels “positive” versus “negative” resources are used in the present study to denote their functional roles in relation to SWB in the context of this study (i.e., buffering vs. amplifying stress-related risks), rather than fixed or context-independent judgments about trait adaptiveness.

This study aims to investigate the relationships between childhood trauma, personality traits, and SWB during adolescence, with a specific focus on differences between early and late adolescence. Using network analysis as the primary method, the study seeks to uncover how these variables interact and differ in their connectivity across developmental stages. By employing network comparison tests, this research will explore variations in network structure, global strength, and edge strength, providing novel insights into the mechanisms through which early life stress shapes personality and SWB during adolescence.

Based on previous studies, three hypotheses are proposed in this study:

#### H1

Adolescent SWB is negatively associated with childhood trauma and neuroticism, but positively associated with agreeableness, openness to experience, conscientiousness, and extraversion.

#### H2

Childhood trauma is connected to well-being both directly and indirectly through its links with personality traits in the network model.

#### H3

The connections between childhood trauma, personality traits, and SWB are expected to differ significantly between early and late adolescence in the network comparison test.

## Methods

### Participants

Data for this study were collected via a questionnaire survey of Chinese adolescents. Participants were conveniently sampled from two public secondary schools in Chongqing, China. There were 2630 valid participants aged between 12 and 18, including 1560 females and 1070 males (*M* = 15.56, *SD* = 1.62), For network comparison, the sample was divided into two developmental stages: early adolescence (ages 12–15, *n* = 1254) and late adolescence (ages 16–18, *n* = 1376).

### Procedure

All procedures were conducted in accordance with relevant institutional guidelines and the Declaration of Helsinki. The study was approved by the Institutional Review Board (Approval No. XL-20250724-0001). In coordination with the schools, parents or legal guardians were first informed of the study and their consent was obtained. Subsequently, students completed the online questionnaire via the Wenjuanxing platform, after providing their own assent. The entire process, including consent and survey completion, took approximately 15 min.

### Measures

#### Chinese Big Five Personality Inventory Brief Version (CBF-PI-B)

A short version of the Chinese Big Five Personality Questionnaire^77^was used to measure subjects’ personality traits; this scale has been used several times and has good credibility in studies (e.g^78,79^.,. The questionnaire consists of 40 items scored on a 6-point Likert scale ranging from 1 = “very inaccurate” to 6 = “very accurate”. The CBF-PI-B contains 5 subscales focusing on the Big Five personality dimensions: openness, conscientiousness, extraversion, agreeableness, and neuroticism. There are seven reverse-rated items on the CBF, and each intrinsic personality trait is measured using eight items. The Cronbach’s alpha coefficients for the five dimensions of the scale are reported as follows: Neuroticism (α = 0.881), Conscientiousness (α = 0.834), Agreeableness (α = 0.772), Openness (α = 0.843), and Extraversion (α = 0.814).

#### Rosenberg Self-Esteem Scale (RSES)

The Chinese version of the RSES^80,81^ was used to measure subjects’ self-esteem. The scale consists of 10 items, including 5 positive and 5 negative scoring questions, on a 4-point Likert scale ranging from “strongly agree” (1) to “strongly disagree” (4), with a total possible score of between 10 and 40, with higher scores indicating a higher the level of self-esteem. The alpha coefficient of the scale in this study was 0.81.

#### Childhood Trauma Questionnaire-Short Form (CTQ-SF)

The Chinese version of the Childhood Trauma Questionnaire (CTQ), developed by^82^ and revised by^83^, was used to measure the experience of childhood trauma. The scale is one of the world’s most recognized instruments for measuring childhood trauma. The CTQ-SF has 28 items, including five dimensions: emotional abuse, physical abuse, sexual abuse, emotional neglect, and physical neglect. Scored on a 5-point Likert scale from “never” (1) to “always” (5), each subscale ranges from 5 to 25, with a total CTQ-SF score of 25–125. A higher CTQ-SF score indicates more severe childhood trauma. The Cronbach’s alpha coefficients for the five dimensions of the scale are reported as follows: Physical Neglect (α = 0.511), Emotional Neglect (α = 0.854), Emotional Abuse (α = 0.768), Physical Abuse (α = 0.791), and Sexual Abuse (α = 0.791).

#### Index of Well-Being

The Index of Well-Being^84^ was used to measure subjective well-being (SWB). The index consists of nine items, the first eight of which are the Index of General Affect (IGA), measured on a 7-point semantic differential scale between opposing adjectives, and the ninth is a single question overall life satisfaction questionnaire (LSQ). To ensure that higher scores indicate higher well-being, four IGA items (‘interesting-boring’, ‘worthwhile-useless’, ‘hopeful-despairing’, and ‘rewarding-disappointing’) were reverse-scored. The composite Index of Well-Being was calculated according to the original formula^84^: Index of Well-Being = 1.1 × (LS score) + 1.0 × (IGA total score). The alpha coefficient for this scale in this study was 0.92.

### Statistical analysis

Descriptive statistics were calculated using SPSS 22. R software was used to measure Spearman correlation matrices, which were applied due to violations of bivariate normality, and to perform network analysis. We used the *qgraph*^85^ and *glasso*^86^ software packages for estimating and visualizing the network. The Gaussian graphical model (GGM) was chosen as the estimation model, where the edges between nodes and nodes are presented. The thickness of the edges depends on the strength of the correlation between the variables. The blue line indicates a positive correlation, while the red line indicates a negative correlation. In addition, *qgraph* was used to calculate the centrality index, which includes strength, betweenness, and closeness^68^. These three dimensions show the relative importance of each node in the network, with higher values implying higher centrality^69^. Strength centrality is calculated as the sum of direct connections of each node. Intermittency centrality is the shortest path through each node, while proximity centrality measures the sum of the shortest path lengths between the investigated node and the other nodes in the network model. The *mgm* software package was used to estimate the predictability of nodes and to display the common variance between each node and its neighboring nodes, which includes an absolute measure of its interconnectedness^67^. In addition, to measure the accuracy and stability of the network model, the *bootnet* package was employed in this study. To test the accuracy of the edge weights, we bootstrapped with 95% confidence intervals (CIs). The narrower the CIs, the more accurate the estimation. We used the case-discard bootstrap method to test the stability of the network model. Stability is obtained by the correlation stability coefficient (CS-coefficient). The CS-coefficient is unsatisfactory when it is below 0.25, and relatively satisfactory when it is above 0.5. The *NetworkComparisonTest* package was used to test the differences between networks in the two stages of adolescence^70^.

## Results

### The network model in the total sample of adolescence

#### Descriptive statistic and correlations

**Fig. 1:**
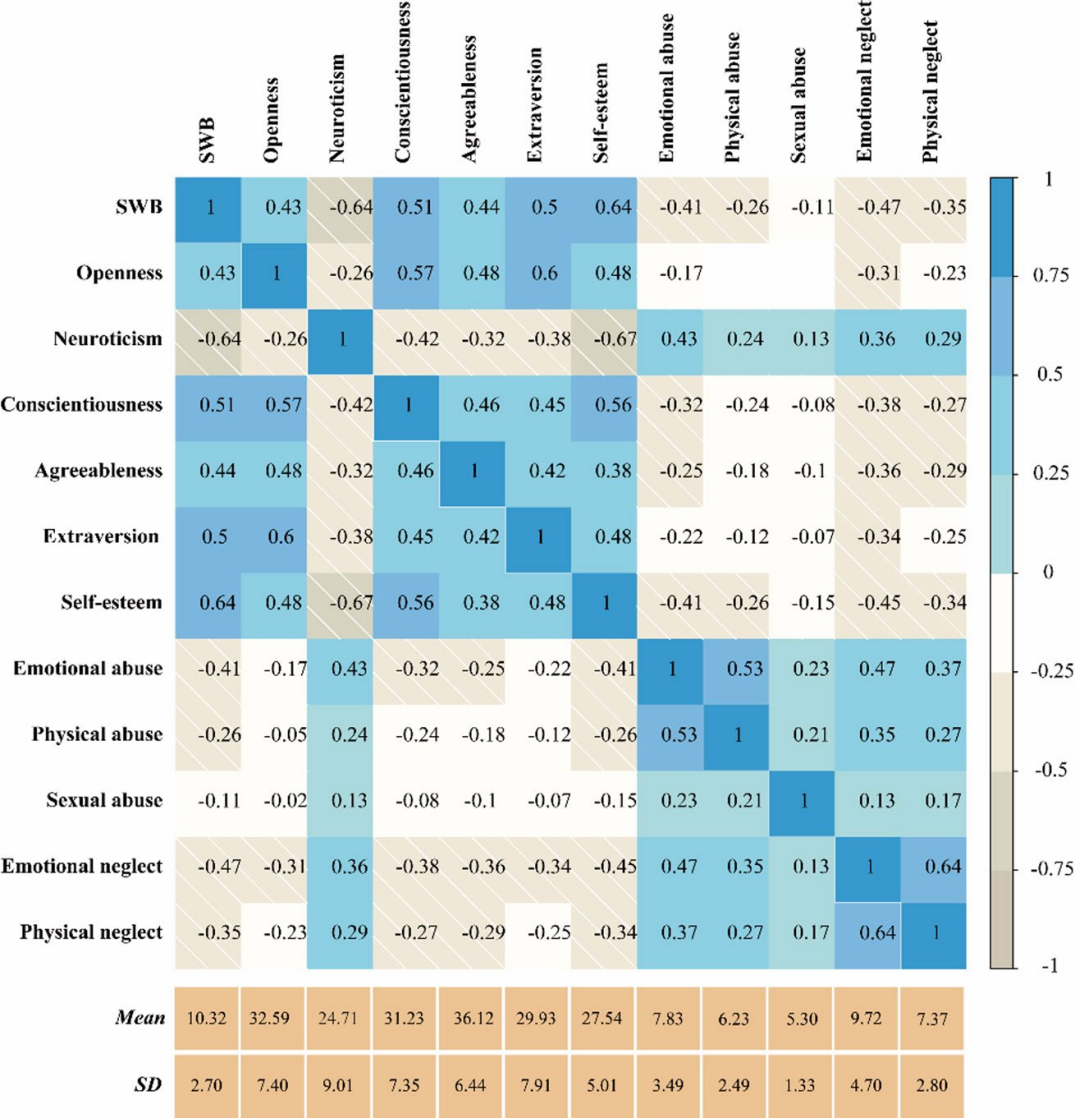
Heatmap of the Spearman correlation matrix. The number in each box is the Spearman correlation coefficient. The lower triangle presents all the coefficients whether significant or not. Only significant coefficients after Bonferroni correction test (*p* < 0.05/(12*11/2)) are displayed in the upper triangle and the blank box refers to nonsignificant.

 Figure 1 presents the mean, standard deviation, and Spearman correlation coefficients for each variable in the study. As shown in the upper triangular section of the heatmap correlation matrix, after applying the Bonferroni correction test (*p* < 0.05/(12 × 11/2)), the results indicate that SWB is positively associated with openness, conscientiousness, agreeableness, and self-esteem, while negatively associated with neuroticism, emotional abuse, physical abuse, sexual abuse, emotional neglect, and physical neglect. These findings provide support for Hypothesis 1 (H1).

#### Network estimation

**Fig. 2:**
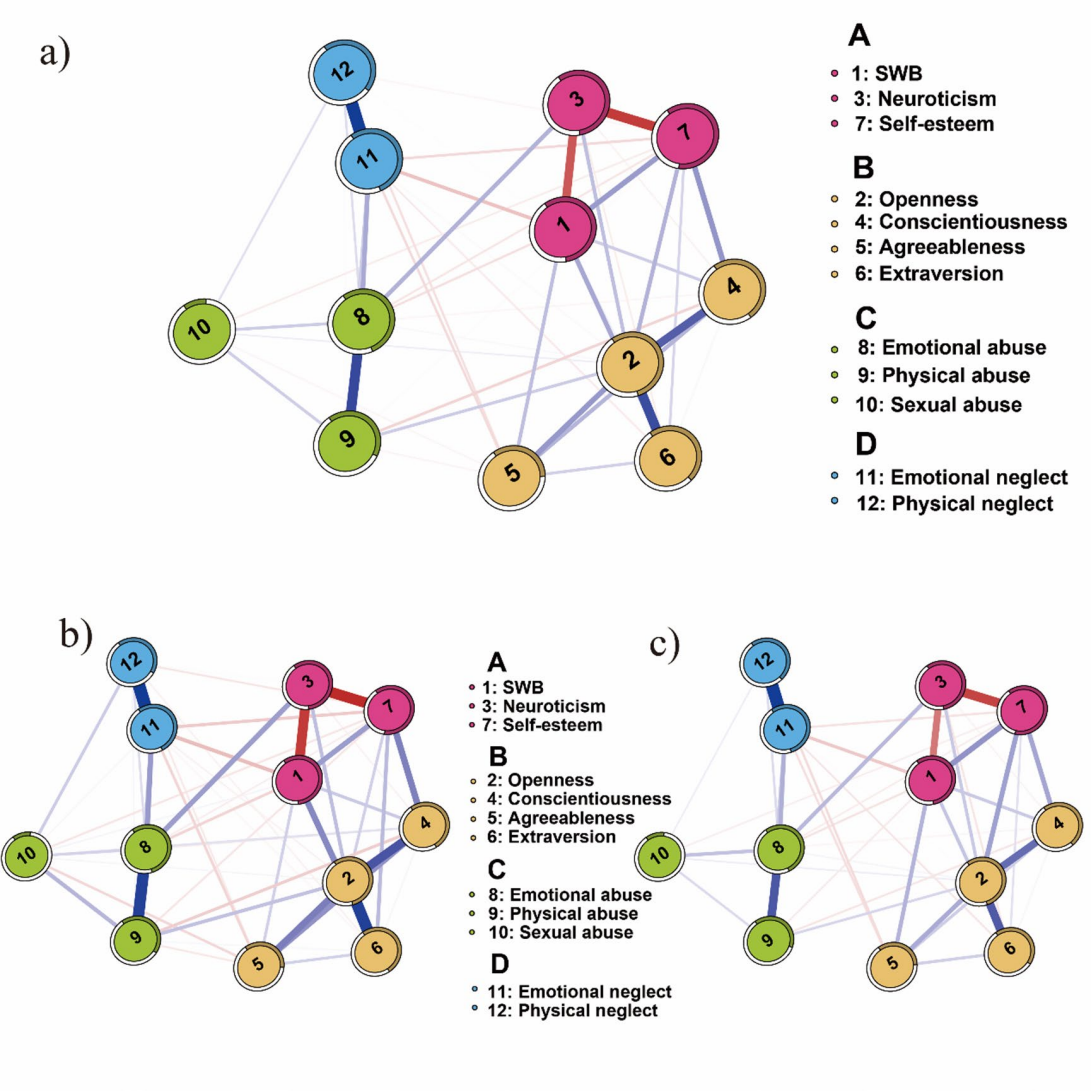
Network Model of Childhood Trauma, Personality, and SWB. Blue lines represent positive connections, red lines represent negative connections. The thickness of lines represents the strength of connections. (**a**) represents the network model of total adolescence sample (*n* = 2630); (**b**) illustrates the network model of early adolescence sample (ages 12–15, *n* = 1254); (**c**) depicts the network model of late adolescence sample (ages 16–18, *n* = 1376).

Figure 2a illustrates the network model of childhood trauma, personality, and subjective well-being (SWB) for the total adolescence sample, comprising 12 nodes. Within the domain of personality and childhood trauma, the negative aspect of personality, neuroticism, is linked solely to emotional abuse (*r* = 0.14). Meanwhile, among positive personality resources, openness shows a positive connection with physical abuse (*r* = 0.09) and a weak and negligible connection with sexual abuse (*r* = 0.03). Conscientiousness displays negative connections with both physical abuse and emotional neglect (*r* = −0.08 and *r* = −0.04, respectively), while agreeableness is negatively associated with emotional neglect (*r* = −0.07) and physical neglect (*r* = −0.06). Extraversion, similarly, has a negative connection with emotional neglect (*r* = −0.04). Self-esteem has negative connections with both emotional neglect (*r* = −0.07), emotional abuse (*r* = −0.05), and sexual abuse (*r* = −0.04).

Among all non-zero edges linked to SWB, negative personal resource such as neuroticism had the strongest connection with SWB (*r* = − 0.33). Among positive personal resources, the connection between SWB and self-esteem is the strongest (*r* = 0.19), followed by extraversion (*r* = 0.16), agreeableness (*r* = 0.12), and conscientiousness (*r* = 0.09). The weakest connection with SWB is openness (*r* = 0.03). Regarding the relationship between SWB and childhood trauma, SWB has the strongest connection with emotional neglect (*r* = −0.11), followed by the connection between SWB and emotional abuse (*r* = −0.06). There is no connection between SWB and physical neglect, physical abuse, or sexual abuse. Notably, in contrast to the moderate-degree correlations observed in the matrix, the network model reveals weaker or absent connections between childhood trauma and subjective well-being, indicating that their relationship is likely mediated by personality traits. These findings support our H2.

The Spinglass algorithm identified four communities out of 12 nodes. Cluster A, which includes SWB, neuroticism, and self-esteem, indicates that SWB is most closely associated with these two dimensions of personality resources. Cluster B includes openness, conscientiousness, agreeableness, and extraversion, all of which are parts of the positive personality resources. Cluster C consists of nodes for emotional abuse, physical abuse, and sexual abuse, all of which are dimensions related to abuse within childhood trauma. Lastly, Cluster D includes emotional neglect and physical neglect, which are the two dimensions related to neglect within childhood trauma.

As shown in Table 1, the predictability of node SWB was 0.58, indicating that 58% of its variance could be explained by surrounding nodes. The mean node predictability was 0.47, meaning that, on average, 47% of each node’s variance was explained by its neighbors in the network model.

#### Centrality estimation

**Fig. 3:**
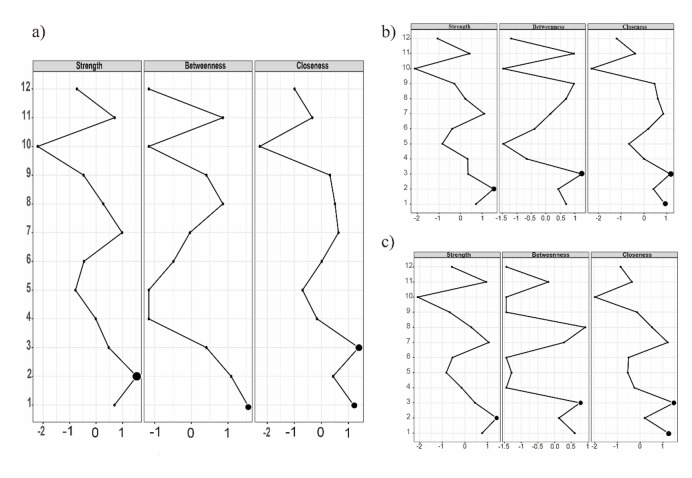
Centrality plots for 12 variables in the network model. The X-axis indicates the standardized z-score of three centrality indices (strength, betweenness, and closeness). The higher the value is, the more central the node is. Each number on the Y-axis represents a variable, as follows: 1: SWB, 2: Neuroticism, 3: Self-esteem, 4: Openness, 5: Conscientiousness, 6: Agreeableness, 7: Extraversion, 8: Emotional abuse, 9: Physical abuse, 10: Sexual abuse, 11: Emotional neglect, 12: Physical neglect. (**a**) represents the network model of total adolescence sample; (**b**) illustrates the network model of early adolescence sample (ages 12–15); (**c**) depicts the network model of late adolescence sample (ages 16–18).

Figure 3a presents the centrality of each node in the network model for the total sample. Larger dots represent nodes with stronger centrality indices. Node 2 (“Openness”) had the greatest impact on the individual, reflecting its highest strength centrality. Node 1 (“SWB”) exhibited the highest closeness centrality, indicating its effects spread most rapidly to other nodes. Nodes 1 (“SWB”) and 3 (“Neuroticism”) demonstrated the highest betweenness centrality, functioning as key bridges that connect different nodes within the network. For more details, see Table 1.

#### Network accuracy and stability

The edge-weight bootstrapping results (Fig. A1a) indicate small confidence intervals (CIs), suggesting relatively precise edge weight estimates. The CS coefficient (Fig. A2a) demonstrates good network stability, with strength = 0.75, closeness = 0.75, and betweenness = 0.21. The bootstrapped difference test revealed that the strongest edges significantly differed from the weakest (Fig. A3a), and the highest centrality indices were distinct from the lowest (Fig. A4a).

**Table 1:** List of nodes, their predictability, and their centrality estimation.

Nodes	Predictability (*R*^2^)			Strength			Betweenness			Closeness		
	(a)	(b)	(c)	(a)	(b)	(c)	(a)	(b)	(c)	(a)	(b)	(c)
1 SWB	0.58	0.59	0.57	0.69	0.72	0.76	1.54	0.70	1.10	1.19	0.91	1.24
2 Openness	0.55	0.56	0.55	1.53	1.53	1.40	1.09	0.43	0.63	0.43	0.41	0.20
3 Neuroticism	0.58	0.58	0.57	0.48	0.35	0.45	0.41	1.25	1.26	1.37	1.15	1.48
4 Conscientiousness	0.50	0.51	0.49	− 0.01	0.33	− 0.15	− 1.16	− 0.66	− 0.94	− 0.16	0.00	− 0.26
5 Agreeableness	0.34	0.36	0.34	− 0.78	− 0.84	− 0.83	− 1.16	− 1.47	− 0.79	− 0.70	− 0.66	− 0.54
6 Extraversion	0.47	0.50	0.44	− 0.46	− 0.39	− 0.55	− 0.49	− 0.39	− 0.94	0.01	0.19	− 0.51
7 Self− esteem	0.62	0.62	0.64	0.98	1.10	1.06	− 0.04	0.16	0.79	0.63	0.84	1.22
8 Emotional abuse	0.54	0.55	0.53	0.27	0.22	0.28	0.86	0.70	1.42	0.50	0.60	0.52
9 Physical abuse	0.42	0.44	0.40	− 0.47	− 0.28	− 0.68	0.41	0.97	− 0.94	0.32	0.46	− 0.14
10 Sexual abuse	0.11	0.12	0.12	− 2.20	− 2.11	− 2.09	− 1.16	− 1.47	− 0.94	− 2.27	− 2.31	− 1.99
11 Emotional neglect	0.54	0.52	0.55	0.70	0.42	0.94	0.86	0.97	0.31	− 0.34	− 0.39	− 0.36
12 Physical neglect	0.45	0.43	0.47	− 0.73	− 1.06	− 0.58	− 1.16	− 1.20	− 0.94	− 0.99	− 1.20	− 0.86

### The network model in the early and late adolescence

#### Network estimation

Figure 2b illustrates the network model for the early adolescence sample, featuring 12 nodes. Within personality and childhood trauma, neuroticism shows a strong association with emotional abuse (*r* = 0.16). Among positive personality traits, openness is linked solely to physical abuse (*r* = 0.10). Conscientiousness is negatively associated with emotional abuse, physical abuse, and emotional neglect (*r* = −0.03, *r* = −0.08, and *r* = −0.03, respectively) but positively correlated with sexual abuse (*r* = 0.05). Agreeableness had negative connections with physical abuse, sexual abuse, physical neglect, and emotional neglect (*r* = −0.04, *r* = −0.06, *r* = −0.07, and *r* = −0.03, respectively). Similarly, self-esteem has negative correlations with physical abuse, sexual abuse, physical neglect, and emotional neglect (*r* = −0.04, *r* = −0.04, *r* = −0.09, and *r* = −0.04, respectively). Extraversion, however, shows no connections with any dimension of childhood trauma.

Among all non-zero edges linked to SWB, neuroticism exhibits the strongest negative association (*r* = −0.34). For positive traits, extraversion has the strongest connection to SWB (*r* = 0.20), followed by self-esteem, conscientiousness, and agreeableness (*r* = 0.16, *r* = 0.09, and *r* = 0.08, respectively). Openness has the weakest association (*r* = 0.03). Regarding childhood trauma, SWB is most strongly connected with emotional neglect (*r* = −0.11), followed by emotional abuse (*r* = −0.07). There are no connections between SWB and physical neglect, physical abuse, or sexual abuse.

Figure 2c presents the network model for late adolescence, also with 12 nodes. Neuroticism remains significantly associated with emotional abuse (*r* = 0.14). Among positive traits, openness is positively correlated with physical abuse and sexual abuse (*r* = 0.08 and *r* = 0.03, respectively). Conscientiousness shows negative associations with physical abuse and emotional neglect (*r* = −0.05 for both). Agreeableness is negatively linked solely to physical neglect and emotional neglect (*r* = −0.06 and *r* = −0.07, respectively). Self-esteem is negatively associated with emotional abuse, sexual abuse, and emotional neglect (*r* = −0.04, *r* = −0.03, and *r* = −0.05, respectively). Extraversion is negatively linked only to emotional neglect (*r* = −0.05).

In late adolescence, neuroticism continues to have the strongest negative association with SWB (*r* = −0.30). Among positive traits, self-esteem shows the strongest connection to SWB (*r* = 0.22), followed by agreeableness, extraversion, and conscientiousness (*r* = 0.15, *r* = 0.12, and *r* = 0.11, respectively). Openness, however, shows no significant association. Regarding childhood trauma, SWB is most strongly linked with emotional neglect (*r* = −0.11), followed by emotional abuse and physical abuse (*r* = −0.06 and *r* = −0.04, respectively). No significant associations are observed between SWB and physical neglect or sexual abuse.

In the network model for the early adolescence sample (Table 1b), the predictability of the SWB node was 0.59, indicating that 59% of its variance could be accounted for by its surrounding nodes. The average predictability across all nodes was 0.48, suggesting that, on average, 48% of the variance for each node was explained by its neighbors within the network. Similarly, in the late adolescence sample, the SWB node also exhibited a predictability of 0.59, with 59% of its variance explained by surrounding nodes. The mean predictability for all nodes in this model was slightly lower at 0.47, indicating that 47% of the variance for each node, on average, was attributable to its neighboring nodes.

#### Centrality estimation

Figure 3b and c illustrate the centrality of each node in the network model for early and late adolescence, respectively. In early adolescence, Node 2 (“Openness”) has the highest strength centrality, while Node 3 (“Neuroticism”) exhibits the highest closeness and betweenness centrality. In late adolescence, Node 2 (“Openness”) continues to have the highest strength centrality, Node 8 (“Emotional Abuse”) demonstrates the highest betweenness centrality, and Node 3 (“Neuroticism”) maintains the highest closeness centrality.

#### Network accuracy and stability

The edge-weight bootstrapping results (Fig. A1b and A1c) indicate moderate accuracy for the adolescent stage networks. The CS coefficients (Fig. A2b and A2c) reveal centrality indices of 0.75 for strength, 0.75 for closeness, and 0.13 for betweenness in early adolescence, and 0.75, 0.67, and 0.52, respectively, in late adolescence. The bootstrapped difference test results are shown in Fig. A3b and A3c, while the node centrality difference test results are displayed in Fig. A4b and A4c.

#### Network comparison

Network comparisons between early and late adolescence were conducted using three tests. The network structure invariance test revealed no significant differences in overall structure (*p* = 0.08), indicating similarity between the two stages. Similarly, the global strength invariance test showed no significant differences in network strength (early = 5.60, late = 5.34; *p* = 0.27). However, the edge invariance test identified significant differences in specific connections. Specifically, eight edges demonstrated significant differences between early and late adolescence.

Among these, the edges linking SWB with agreeableness (*p* = 0.03) and physical abuse (*p* = 0.004) were weaker in early adolescence (*r* = 0.08, *r* = 0) compared to late adolescence (*r* = 0.15, *r* = −0.04). Conversely, the edge between SWB and extraversion (*p* = 0.03) was stronger in early adolescence (*r* = 0.19) than in late adolescence (*r* = 0.12). Additionally, connections between conscientiousness and agreeableness (*p* = 0.03) and conscientiousness and sexual abuse (*p* = 0.002) were stronger in early adolescence (*r* = 0.18, *r* = 0.05) compared to late adolescence (*r* = 0.10, *r* = −0.02). In contrast, the edge between openness and self-esteem (*p* = 0.006) was weaker in early adolescence (*r* = 0.08) than in late adolescence (*r* = 0.19). Further differences included a stronger connection between agreeableness and sexual abuse (*p* = 0.01) in early adolescence(*r* = −0.06) compared to late adolescence (*r* = 0). Lastly, the link between emotional neglect and physical neglect (*p* = 0.004) was weaker in early adolescence (*r* = 0.45) than in late adolescence (*r* = 0.55). To summarize, these findings support our H3.

## Discussion

The present study used a network approach to examine the stress process model, investigating how childhood trauma, as a chronic stressor, relates to adolescent SWB through the mediation of personal resources, namely personality traits and self-esteem. The findings revealed that childhood trauma, as a chronic stressor, hinders the development of positive personal resources while fostering negative traits, particularly neuroticism, which significantly impacts SWB in adolescents. While the overall network structure and global strength were similar across early and late adolescence, notable differences in specific edge connections were observed between the two groups, highlighting developmental variations in these relationships.

### Personality traits as key determinants of SWB

Our results revealed that SWB exhibited a negative connection with negative personal resources, such as neuroticism, and positive connections with positive personal resources, including self-esteem, agreeableness, extraversion, conscientiousness, and openness, within the total adolescence sample. Self-esteem emerged as the strongest positive predictor of SWB in our network models, with its influence becoming even more pronounced in late adolescence. However, some specific connections demonstrated notable group differences between early and late adolescence stages.

Individuals with high neuroticism tend to focus excessively on negative events, leading to heightened negative emotions^48,49^. Additionally, neuroticism is characterized by heightened sensitivity to threats^87^, which adversely impacts psychological well-being and, consequently, SWB. In contrast^88^, highlighted that individuals who are “extraverted, optimistic, worry-free,” possess high self-esteem, and maintain moderate aspirations tend to experience greater happiness. Extraverted individuals are adept at interacting with others and maintaining a positive outlook when facing challenges, which explains the strong association between extraversion and SWB.

Similarly, individuals high in agreeableness and conscientiousness are more likely to receive positive feedback and reinforcement from others, fostering a higher sense of SWB^38^. Therefore, both agreeableness and conscientiousness are positively linked to SWB. On the other hand, openness shows a relatively weaker association with SWB^64^. These positive personality traits function as individual assets that interact with external environments to create developmental assets, such as support, empowerment, boundaries and expectations, and constructive use of time. Together, these individual and developmental assets act as internal and external factors that influence well-being, working in tandem to promote positive development and SWB^89,90^.

Interestingly, some developmental trends emerged between the groups. Although neuroticism consistently showed strong negative connections with SWB across stages, its strength slightly weakened from early to late adolescence, possibly reflecting improved emotional regulation over time^91^, even though this trend was not statistically significant. Meanwhile, self-esteem emerged as the strongest positive predictor of SWB, with its influence becoming more pronounced in late adolescence. This change, though not statistically significant, may underscore the growing importance of self-concept and identity development at this stage^92^. Extraversion contributed more strongly to SWB in early adolescence, likely due to the developmental focus on forming peer connections and building social networks^93^. Extraverted individuals, being outgoing and socially active, are better able to connect with others and adapt to these social demands, which may directly improve their well-being. In contrast, agreeableness gained greater relevance in late adolescence, a period marked by the increasing importance of deeper social bonds and cooperative interactions^94^. During this stage, adolescents face more complex interpersonal relationships, such as preparing for adulthood and fostering trust and understanding in social and academic contexts. Traits like kindness and empathy, characteristic of agreeableness, likely facilitate these transitions, strengthening its association with SWB in late adolescence.

In summary, this study substantiates and elaborates on the stress process model by revealing that the pathways from childhood trauma to SWB are not uniform. The influence of specific personal resources evolves during adolescence, suggesting a developmentally-sensitive version of the model where the salience of certain resources changes with developmental tasks. Neuroticism’s negative impact weakens slightly over time, while self-esteem becomes increasingly important in late adolescence. Extraversion strongly supports SWB in early adolescence through social engagement, whereas agreeableness gains relevance later, reflecting the growing need for deeper social connections.

### Emotional abuse and emotional neglect linked to SWB via personality

Our research identified emotional abuse as the only dimension of childhood trauma consistently linked to heightened neuroticism across all adolescent stages. This suggests that individuals exposed to emotional abuse during childhood may develop greater emotional sensitivity and difficulty managing negative emotions, core traits of neuroticism^95^. While this heightened reactivity might initially serve as an adaptive response to unstable or threatening environments, over time, it likely contributes to persistent emotional distress and vulnerability to negative psychological outcomes^96^, ultimately impairing subjective well-being (SWB)^97^.

Emotional neglect was consistently associated with lower conscientiousness, agreeableness, extraversion, and self-esteem across all stages of adolescence, underscoring its pervasive impact on personality development and self-perception. The lack of emotional support may disrupt the development of responsibility and goal-oriented behaviors, diminishing conscientiousness^98^. Similarly, the lack of warmth and trust likely suppresses empathy and cooperation^99^, further impacts agreeableness^100^, while discouraging social engagement and assertiveness, which may reduce extraversion. Emotional neglect also undermines self-worth, fostering feelings of inadequacy and leading to lower self-esteem^101^. These enduring negative associations emphasize how early emotional neglect shapes maladaptive personality traits and self-concept, further diminishes SWB.

Interestingly, openness demonstrated consistent positive connections with physical abuse across all adolescent stages. Physical abuse may heighten awareness and foster introspection as adaptive coping mechanisms, which could later manifest as traits characteristic of openness, such as imaginative thinking and curiosity about diverse perspectives^99^. Despite this, openness showed only a weak direct association with SWB, suggesting its impact on well-being may be mediated by other traits, particularly self-esteem.

We also observed significant group differences in the connection between openness and self-esteem, with a weaker association in early adolescence compared to late adolescence. During early adolescence, identity exploration and self-concept are still developing, limiting the extent to which openness may enhance self-esteem because research had consistently found openness and self-esteem had strong correlations^102,103^. In late adolescence, however, greater life experience and a clearer sense of identity appear to strengthen the role of openness in fostering self-esteem, which may further improve SWB. In contrast, other forms of trauma, such as physical and sexual abuse, demonstrated weaker connections in the network. This may indicate that their impact on adolescent SWB is less direct, potentially operating through other mediating mechanisms (e.g., specific psychopathology) not fully captured here, or that emotional maltreatment represents a core dimension with more pervasive effects on personality development.

### Implications and limitations

This study identified the influencing paths of childhood trauma, personality, and SWB. Childhood trauma serves as a social environment that shapes individuals’ personality traits, thereby influencing their SWB. This network model directly presents the interrelationship between childhood trauma, personality traits, and SWB, from which it was revealed that the closest construct to SWB is personality. Neuroticism is a personality trait that negatively affects adolescent SWB, highlighting the value of recognizing vulnerability associated with neuroticism and supporting strengths and psychosocial resources (e.g., openness, agreeableness, conscientiousness, extraversion, and self-esteem) that are linked to better SWB. Childhood trauma is a factor that is negatively correlated with personality and has an impact on SWB via personality. That is, childhood trauma may be a negative factor of SWB, but not all adolescents with childhood trauma will have lower SWB. Therefore, the personality traits of adolescents are important in worsening or improving their SWB.

In addition, there are some limitations to this study. First, longitudinal network analyses are needed to verify the directionality and potential causal dynamics between these variables, such as testing whether neuroticism prospectively predicts decreases in SWB. Future studies could also expand the network by incorporating other critical mechanisms like social support, specific emotion regulation strategies, or psychopathological symptoms to build a more comprehensive ecological model. Second, the measures in this study, including the Childhood Trauma Questionnaire (CTQ), were self-reported. This is a common limitation in trauma research, as feelings of shame, guilt, or denial might introduce underreporting bias, potentially affecting the accuracy of the findings^17,104^. Furthermore, cross-group comparisons in clinical or cross-cultural samples could test the generalizability of these networks and identify unique pathways in specific populations. Cultural contexts may shape how childhood trauma is interpreted and reported, as well as the social value and functional roles of specific personality traits and self-esteem, which in turn may alter the relative strength or salience of pathways linking trauma, personality, and subjective well-being^105–108^. Moreover, cultural meaning systems can guide how adversity is appraised and integrated into the self. In East Asian contexts, more positive beliefs about adversity have been linked to better psychological adjustment and well-being^109,110^. Cross-cultural evidence also suggests that East Asian individuals may be more likely than Western individuals to derive meaning from stressful experiences and to adopt positive reframing coping styles^111^. These culturally shaped interpretations may moderate whether and how trauma-related experiences translate into personal resources and SWB, suggesting that future research should explicitly test cultural differences in the trauma–resources–SWB pathways. Ultimately, the central nodes identified here, such as neuroticism and self-esteem, provide empirically-derived targets for developing and testing the efficacy of tailored psychological interventions, moving from network models to targeted intervention design and evaluation in applied settings.

## Conclusion

This study identified the pathways through which childhood trauma, as a chronic stressor, influences adolescent subjective well-being (SWB) via personality traits, with neuroticism and self-esteem emerging as the most significant factors in maintaining or improving SWB. Developmental stage-specific differences were observed in the connections between personality traits, particularly extraversion and agreeableness, and SWB. Additionally, differences were found in the interactions among personality traits, especially between openness and self-esteem. These findings suggest that developmental differences in personality play a crucial role in shaping adolescent SWB. While emotional abuse and neglect had the pivotal influence on shaping personality traits.

## Supplementary Information

Below is the link to the electronic supplementary material.

Supplementary Material 1

## Data Availability

The datasets are available from the corresponding author on reasonable request.
