# Supplementary material for: The relationship between childhood trauma, personality, and subjective well-being in early and late adolescence: a network analysis
Source: Sci Rep. 2026 Feb 26;16:8870. doi: 10.1038/s41598-026-41659-0 (PMC12988061; doi:10.1038/s41598-026-41659-0)
Supplement: Supplementary file 1 — Supplementary Material 1 [file 41598_2026_41659_MOESM1_ESM.docx]

|  | 1 | 2 | 3 | 4 | 5 | 6 | 7 | 8 | 9 | 10 | 11 | 12 |
| --- | --- | --- | --- | --- | --- | --- | --- | --- | --- | --- | --- | --- |
| 1 | 0.00 | 0.03 | -0.33 | 0.09 | 0.12 | 0.16 | 0.19 | -0.06 | 0.00 | 0.00 | -0.11 | -0.01 |
| 2 | 0.03 | 0.00 | 0.12 | 0.31 | 0.19 | 0.38 | 0.14 | 0.01 | 0.09 | 0.03 | -0.01 | 0.00 |
| 3 | -0.33 | 0.12 | 0.00 | -0.02 | 0.00 | -0.03 | -0.39 | 0.14 | 0.00 | 0.00 | 0.00 | 0.00 |
| 4 | 0.09 | 0.31 | -0.02 | 0.00 | 0.14 | 0.01 | 0.20 | -0.02 | -0.08 | 0.00 | -0.04 | 0.00 |
| 5 | 0.12 | 0.19 | 0.00 | 0.14 | 0.00 | 0.09 | 0.00 | 0.00 | -0.02 | -0.02 | -0.07 | -0.06 |
| 6 | 0.16 | 0.38 | -0.03 | 0.01 | 0.09 | 0.00 | 0.07 | 0.00 | 0.00 | 0.00 | -0.04 | 0.00 |
| 7 | 0.19 | 0.14 | -0.39 | 0.20 | 0.00 | 0.07 | 0.00 | -0.05 | -0.01 | -0.04 | -0.07 | -0.02 |
| 8 | -0.06 | 0.01 | 0.14 | -0.02 | 0.00 | 0.00 | -0.05 | 0.00 | 0.39 | 0.09 | 0.16 | 0.06 |
| 9 | 0.00 | 0.09 | 0.00 | -0.08 | -0.02 | 0.00 | -0.01 | 0.39 | 0.00 | 0.09 | 0.09 | 0.01 |
| 10 | 0.00 | 0.03 | 0.00 | 0.00 | -0.02 | 0.00 | -0.04 | 0.09 | 0.09 | 0.00 | 0.00 | 0.05 |
| 11 | -0.11 | -0.01 | 0.00 | -0.04 | -0.07 | -0.04 | -0.07 | 0.16 | 0.09 | 0.00 | 0.00 | 0.50 |
| 12 | -0.01 | 0.00 | 0.00 | 0.00 | -0.06 | 0.00 | -0.02 | 0.06 | 0.01 | 0.05 | 0.50 | 0.00 |

**Table A1** The edge weight matrix in the network model of total sample

1= subjective well-being (SWB), 2=openness, 3=neuroticism, 4=conscientiousness, 5=agreeableness, 6=extraversion, 7=self-esteem, 8=emotional abuse, 9=physical abuse, 10=sexual abuse, 11=emotional neglect, 12=physical neglect

|  | 1 | 2 | 3 | 4 | 5 | 6 | 7 | 8 | 9 | 10 | 11 | 12 |
| --- | --- | --- | --- | --- | --- | --- | --- | --- | --- | --- | --- | --- |
| 1 | 0.00 | 0.03 | -0.34 | 0.09 | 0.08 | 0.20 | 0.16 | -0.07 | 0.00 | 0.02 | -0.11 | 0.00 |
| 2 | 0.03 | 0.00 | 0.10 | 0.30 | 0.21 | 0.40 | 0.08 | 0.00 | 0.10 | 0.02 | -0.02 | 0.00 |
| 3 | -0.34 | 0.10 | 0.00 | -0.02 | 0.00 | 0.00 | -0.37 | 0.16 | 0.00 | 0.00 | 0.00 | 0.01 |
| 4 | 0.09 | 0.30 | -0.02 | 0.00 | 0.18 | 0.01 | 0.21 | -0.03 | -0.08 | 0.05 | -0.03 | 0.00 |
| 5 | 0.08 | 0.21 | 0.00 | 0.18 | 0.00 | 0.07 | 0.00 | 0.00 | -0.04 | -0.06 | -0.07 | -0.03 |
| 6 | 0.20 | 0.40 | 0.00 | 0.01 | 0.07 | 0.00 | 0.11 | 0.00 | 0.01 | 0.00 | -0.01 | -0.02 |
| 7 | 0.16 | 0.08 | -0.37 | 0.21 | 0.00 | 0.11 | 0.00 | -0.03 | -0.04 | -0.04 | -0.09 | -0.04 |
| 8 | -0.07 | 0.00 | 0.16 | -0.03 | 0.00 | 0.00 | -0.03 | 0.00 | 0.40 | 0.07 | 0.17 | 0.05 |
| 9 | 0.00 | 0.10 | 0.00 | -0.08 | -0.04 | 0.01 | -0.04 | 0.40 | 0.00 | 0.12 | 0.07 | 0.01 |
| 10 | 0.02 | 0.02 | 0.00 | 0.05 | -0.06 | 0.00 | -0.04 | 0.07 | 0.12 | 0.00 | -0.01 | 0.08 |
| 11 | -0.11 | -0.02 | 0.00 | -0.03 | -0.07 | -0.01 | -0.09 | 0.17 | 0.07 | -0.01 | 0.00 | 0.45 |
| 12 | 0.00 | 0.00 | 0.01 | 0.00 | -0.03 | -0.02 | -0.04 | 0.05 | 0.01 | 0.08 | 0.45 | 0.00 |

**Table A2** The edge weight matrix in the network model of early adolescence sample

|  | 1 | 2 | 3 | 4 | 5 | 6 | 7 | 8 | 9 | 10 | 11 | 12 |
| --- | --- | --- | --- | --- | --- | --- | --- | --- | --- | --- | --- | --- |
| 1 | 0.00 | 0.02 | -0.28 | 0.11 | 0.15 | 0.12 | 0.22 | -0.06 | -0.04 | 0.00 | -0.11 | 0.00 |
| 2 | 0.02 | 0.00 | 0.11 | 0.30 | 0.17 | 0.35 | 0.19 | 0.02 | 0.08 | 0.03 | -0.01 | 0.00 |
| 3 | -0.28 | 0.11 | 0.00 | -0.02 | -0.01 | -0.05 | -0.40 | 0.14 | 0.00 | 0.00 | 0.00 | 0.00 |
| 4 | 0.11 | 0.30 | -0.02 | 0.00 | 0.10 | 0.01 | 0.18 | 0.00 | -0.05 | -0.02 | -0.05 | 0.00 |
| 5 | 0.15 | 0.17 | -0.01 | 0.10 | 0.00 | 0.10 | 0.00 | 0.00 | 0.00 | 0.00 | -0.06 | -0.07 |
| 6 | 0.12 | 0.35 | -0.05 | 0.01 | 0.10 | 0.00 | 0.06 | 0.00 | 0.00 | 0.00 | -0.05 | 0.00 |
| 7 | 0.22 | 0.19 | -0.40 | 0.18 | 0.00 | 0.06 | 0.00 | -0.04 | 0.00 | -0.03 | -0.06 | 0.00 |
| 8 | -0.06 | 0.02 | 0.14 | 0.00 | 0.00 | 0.00 | -0.04 | 0.00 | 0.36 | 0.12 | 0.16 | 0.07 |
| 9 | -0.04 | 0.08 | 0.00 | -0.05 | 0.00 | 0.00 | 0.00 | 0.36 | 0.00 | 0.07 | 0.10 | 0.00 |
| 10 | 0.00 | 0.03 | 0.00 | -0.02 | 0.00 | 0.00 | -0.03 | 0.12 | 0.07 | 0.00 | 0.00 | 0.03 |
| 11 | -0.11 | -0.01 | 0.00 | -0.05 | -0.06 | -0.05 | -0.06 | 0.16 | 0.10 | 0.00 | 0.00 | 0.55 |
| 12 | 0.00 | 0.00 | 0.00 | 0.00 | -0.07 | 0.00 | 0.00 | 0.07 | 0.00 | 0.03 | 0.55 | 0.00 |

**Table A3** The edge weight matrix in the network model of late adolescence sample

**
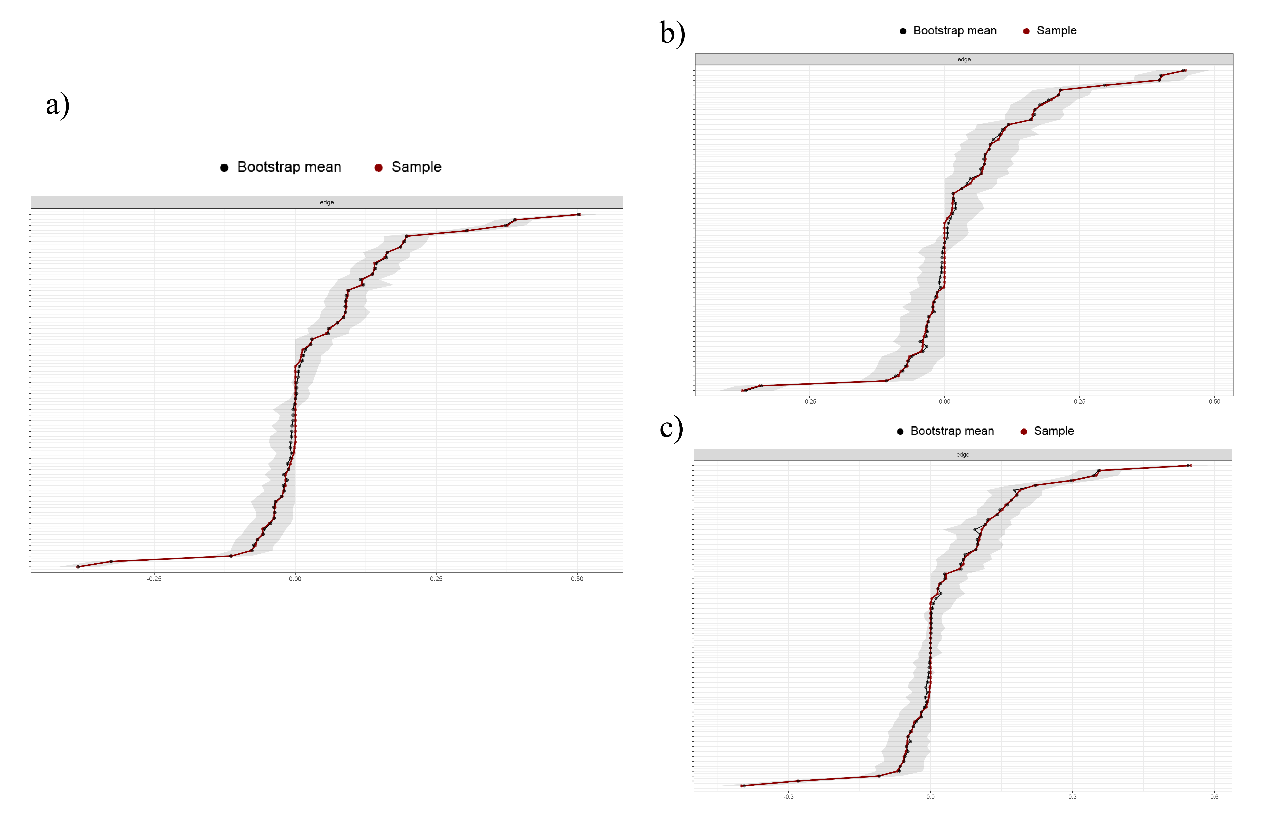
**

**Fig. A1** Bootstrapping of the 95% confidence intervals of the edge weights for the estimated network. The red line indicates the edge weight values, and the gray area indicates the 95% CIs. a) represents the network model of total adolescence sample; b) illustrates the network model of early adolescence sample (ages 12-15); c) depicts the network model of late adolescence sample (ages 16-18)


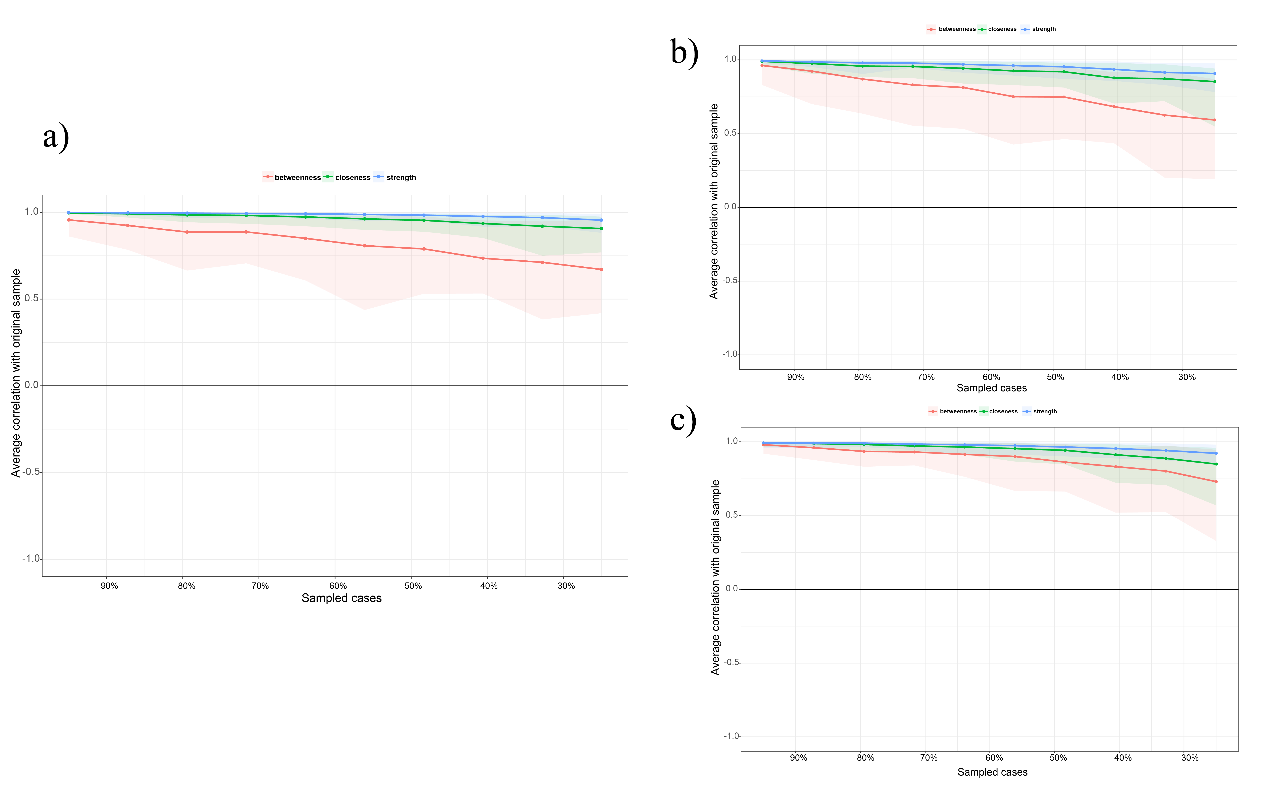


**Fig. A2** Stability of central indices. a) represents the network model of total adolescence sample; b) illustrates the network model of early adolescence sample (ages 12-15); c) depicts the network model of late adolescence sample (ages 16-18)


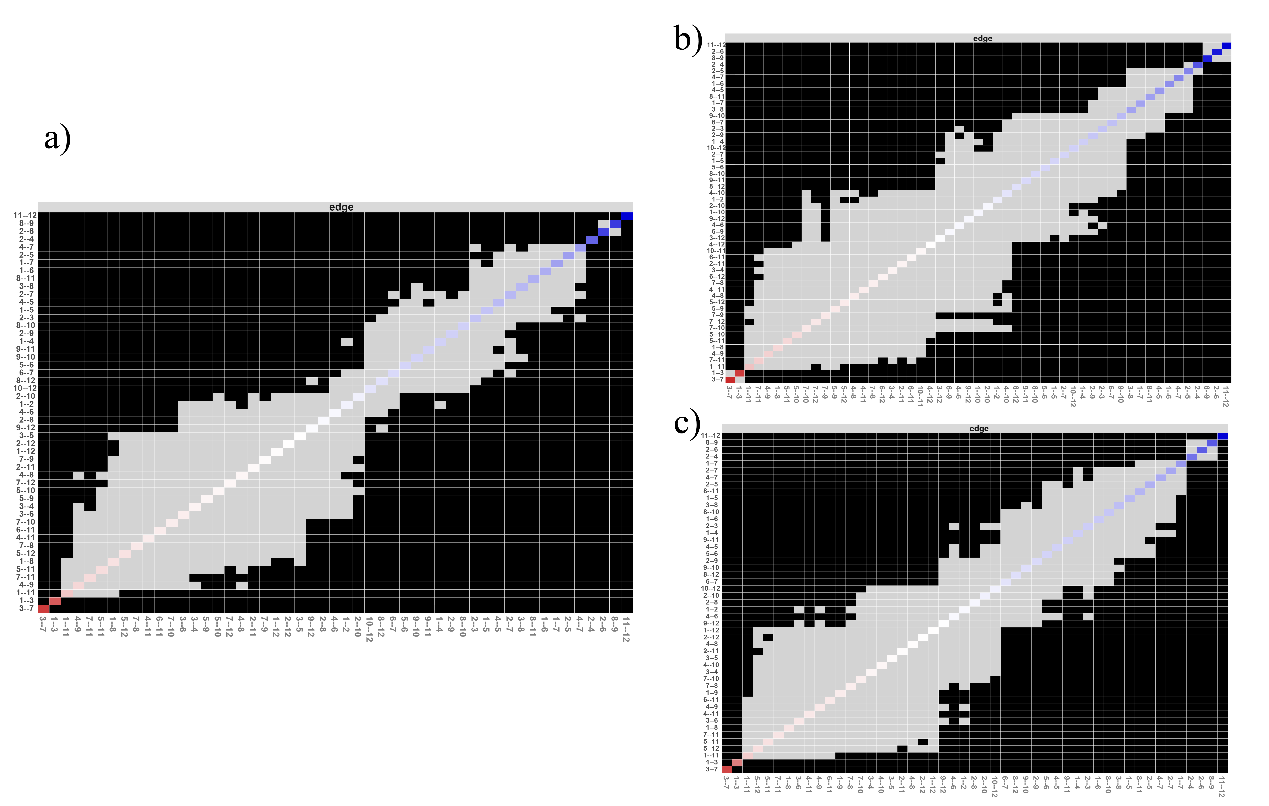


**Fig. A3** Edge weight difference test (α = 0.05) for the estimated network. Gray boxes indicate edges that do not differ significantly from one-another; black boxes represent edges that do differ significantly from one another; colored boxes correspond to the color of the edge in Fig. 2. a) represents the network model of total adolescence sample; b) illustrates the network model of early adolescence sample (ages 12-15); c) depicts the network model of late adolescence sample (ages 16-18)


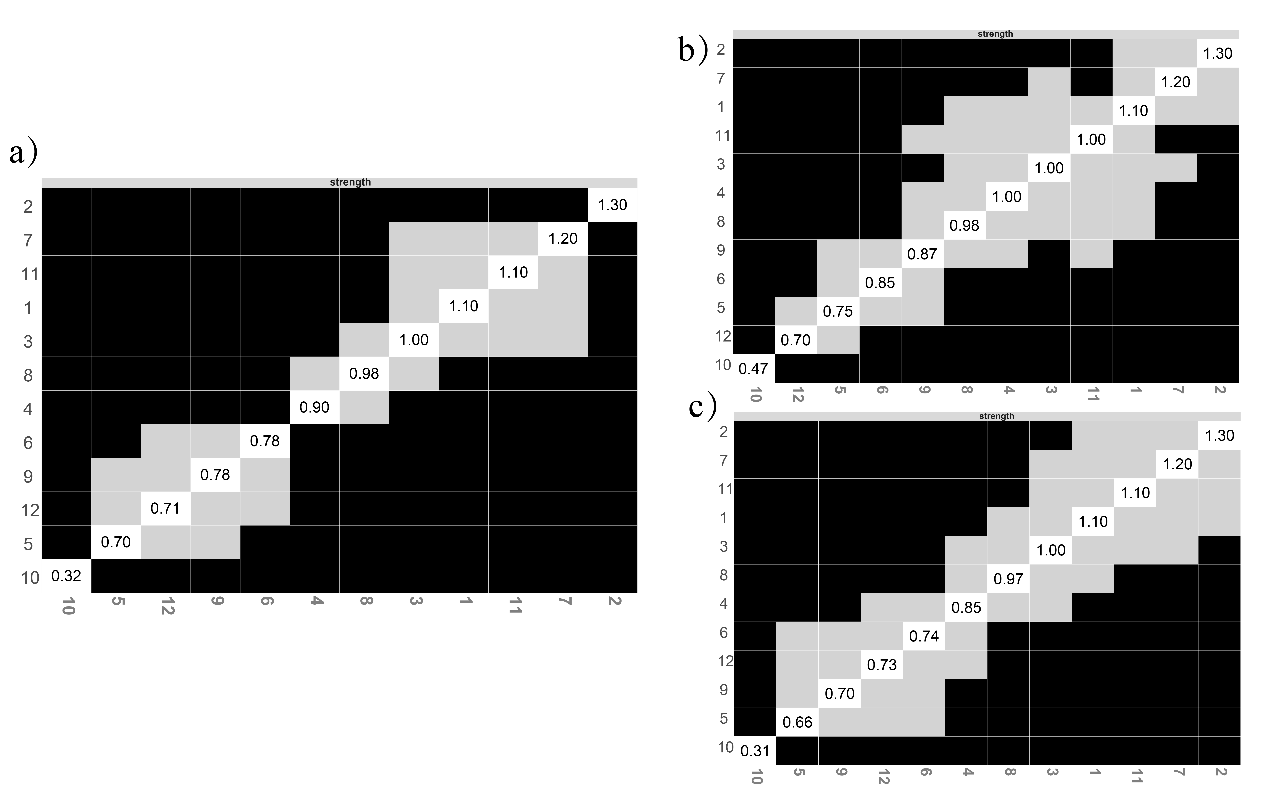


**Fig. A4** Centrality difference test (α = 0.05) for the estimated network. Gray boxes indicate nodes that do not differ significantly from one-another; black boxes represent nodes that do differ significantly from one another; white boxes show the value of node strength. a) represents the network model of total adolescence sample; b) illustrates the network model of early adolescence sample (ages 12-15); c) depicts the network model of late adolescence sample (ages 16-18)
